# The *Nitro-Chloro* Substitution on Two Quinolinone-Chalcones: From Molecular Modeling to Antioxidant Potential

**DOI:** 10.1021/acsomega.5c11439

**Published:** 2026-02-03

**Authors:** Renata Layse G. de Paula, Vitor S. Duarte, Giulio D. C. D’Oliveira, Mirian R. C de Castro, Caridad N. Pérez, Jean M. F. Custódio, Allen G. Oliver, Hamilton B. Napolitano

**Affiliations:** † Grupo de Química Teórica e Estrutural, 425885 Universidade Estadual de Goiás, 75132-903 Anápolis, GO, Brazil; ‡ Department of Chemistry and Biochemistry, 6111 University of Notre Dame, 46556 Notre Dame, Indiana, United States; § Instituto de Química, 67824 Universidade Federal de Goiás, 74690-900 Goiânia, GO, Brazil

## Abstract

The increasing demand for renewable energy has driven the search for sustainable biodiesel additives whose molecular structures exhibit antioxidant potential. This study reports the synthesis and solid-state X-ray diffraction studies of two quinolinone-chalcone derivatives, C_28_H_19_N_3_O_7_S (**QC-NO**_**2**_) and C_28_H_19_ClN_2_O_5_S (**QC-Cl**). Density functional theory calculations and Fukui function analysis were combined with a predictive tool based on previously trained machine learning models to investigate the impact of *nitro* and *chloro* substituents on their physicochemical properties, molecular reactivity, and antioxidant potential. Theoretical results indicated that **QC-Cl** exhibits higher electronic stability, with larger energy gaps (599 kJ/mol) and greater nucleophilicity, while **QC-NO**_**2**_ shows enhanced electrophilicity and electron-accepting ability. Molecular electrostatic potential maps and Fukui functions highlighted reactive sites consistent with the substituent electronic effects, particularly the strong electron-withdrawing character of the nitro group. Predictions of the hydroxyl radical scavenging rate constant (*k*_OH_) obtained using a tool based machine learning models demonstrated that **QC-NO**_**2**_ (6.09 × 10^9^ M^–1^·s^–1^) performs comparably to commercial antioxidants such as BHT and TBHQ. These findings underscore the relevance of structural modification in tuning antioxidant activity and suggest that chalcone-based hybrids, especially **QC-NO**_**2**_, are promising candidates to act as antioxidants in biodiesel.

## Graphical abstract

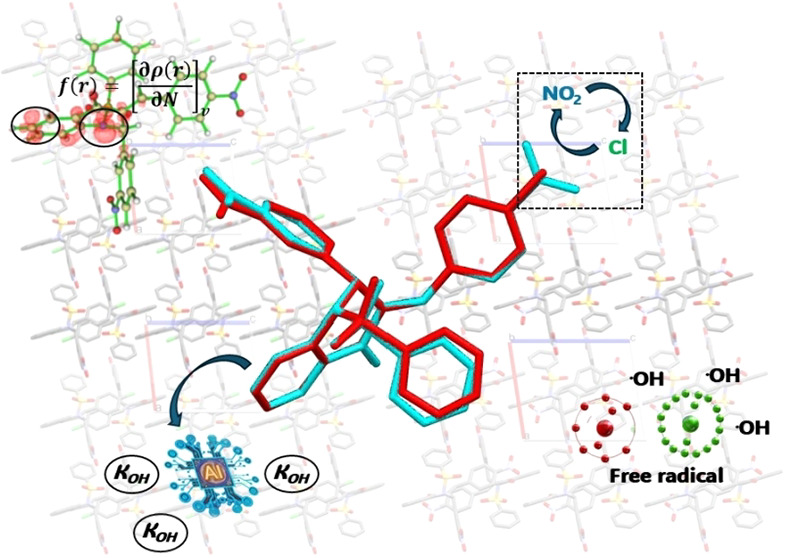

## 1 Introduction

The transition to renewable energy sources has stimulated growing interest in biofuels, particularly biodiesel, as a sustainable alternative to fossil fuels. Biodiesel offers several advantages, including biodegradability, reduced greenhouse gas emissions, and compatibility with existing diesel engines.^1^ However, one of the main limitations to its widespread use is its susceptibility to oxidative degradation during storage and use. Exposure to oxygen, light, and high temperatures favors the formation of free radicals and reactive oxygen species, such as hydroxyl radicals (^•^OH), which initiate and propagate lipid peroxidation reactions.^2,3^ These processes result in the formation of peroxides, acids, and polymeric compounds that compromise fuel quality, reducing its calorific value, and generating deposits capable of damaging engine components.^4^ Therefore, the search for additives with antioxidant potential becomes essential, as controlling oxidation processes is a key requirement for the consolidation of biodiesel in the global energy matrix. One possibility for mitigating oxidative degradation is the use of antioxidant compounds as biodiesel additives. Effective antioxidants act as radical scavengers, preferentially reacting with these species before they attack fatty acid methyl esters (FAMEs), the major constituents of biodiesel.^5,6^ Commercial antioxidants, such as butylhydroxytoluene (BHT), *tert*-butylhydroquinone (TBHQ), and propyl gallate (PG), are widely used in the food and fuel industries. However, issues related to the performance, cost, solubility, and environmental impact of these compounds have motivated the search for new molecules with greater efficiency and selectivity.^7^

In this context, chalcones and their derivatives emerge as promising candidates due to their structural flexibility, relatively simple synthesis, and a wide range of reported biological and chemical properties.^8^ Structural modification in chalcone-derived systems, particularly through hybridization with heterocyclic skeletons such as quinolinones, offers opportunities to tune electronic properties and enhance antioxidant activity.^9,10^ The effect of substituents plays a fundamental role in the stability and reactivity of chalcones and their derivatives. Electronegative groups, such as nitro and chlorine, can alter the energies of frontier molecular orbitals, electrophilicity, and hydrogen bond formation potential, influencing both molecular packing in the solid-state and reactivity toward radical species.^11,12^ Recent studies demonstrate that subtle structural modifications in chalcone-based hybrids can significantly impact their antioxidant capacity, emphasizing the value of integrating experimental and computational approaches in structure–activity based studies.^8,13^

In this work, we analyzed two quinolinone-chalcone derivatives with *nitro* and *chlorine* substituents, respectively: C_28_H_19_N_3_O_7_S (**QC-NO**_**2**_) and C_28_H_19_ClN_2_O_5_S (**QC-Cl**). By combining single-crystal X-ray diffraction, theoretical DFT calculations, and antioxidant activity predictions generated by machine learning based tools, we investigated how these substituents influence molecular conformation, electronic descriptors, and antioxidant potential. Crystallographic analyses revealed that although the compounds have similar molecular conformations, the substituents directly influenced the electronic distribution and crystal packing. DFT calculations confirmed that the nitro group increases electrophilicity and electron-accepting capacity, while chlorine promotes greater electronic stability. Predictions of hydroxyl radical (*k*_OH_) reactivity obtained using a tool based on previously trained machine learning models indicated that **QC-NO**_**2**_ has antioxidant activity comparable to, or superior to, that of commercial standards, while **QC-Cl** demonstrated moderate reactivity. These findings together reinforce the impact of substituent effects on molecular reactivity and suggest **QC-NO**_2_ as a promising antioxidant candidate for biodiesel applications.

## 2 Experimental and Computational Procedures

### 2.1 Synthesis and Solid-State Analysis

**QC-NO**_**2**_ and **QC-Cl** were synthesized according to the methodology described by d’Oliveira et al.^10,14^ The starting chalcone (1.0 mmol) was reacted with 4-nitrobenzaldehyde or 4-chlorobenzaldehyde (2.0 mmol) in basic ethanolic medium at room temperature for 48 h. After filtration and washing with ethanol, the solid was dissolved in dichloromethane, washed with water, and slowly evaporated to afford the products. The synthesis scheme and corresponding spectroscopic characterization of **QC-NO**_**2**_ and **QC-Cl** are provided in Scheme S1 and Figures S1–S8 in the Supporting Information (SI). Single crystals were obtained by recrystallization from dichloromethane using diethyl ether vapor diffusion.

The solid-state analysis for **QC-NO**_**2**_ and **QC-Cl** were determined by single-crystal X-ray diffraction using a Bruker APEX-II CCD diffractometer with Mo Kα radiation (λ = 0.71073 Å). Data were collected at 120 K for **QC-NO**_**2**_ and at 296.15 K for **QC-Cl**. Structure solution and refinement for both compounds were carried out using the SHELX programs,^15^ implemented within the Olex2 software.^16^ Crystallographic data of **QC-NO**_**2**_ and **QC-Cl** were deposited in the Cambridge Crystallographic Data Centre (CCDC)^17^ with CCDC numbers: 2495556 and 2495555, respectively. The intermolecular interactions and supramolecular arrangement were analyzed through geometric features using the Mercury software^18^ and through electron density using Hirshfeld Surfaces (HS) analysis^19–21^ using the CrystalExplorer software,^22^ in addition to the quantification of the molecular interactions present in each molecule through the 2D fingerprint.^23^ The generated HS are based on normalized contact distances, where *de* and *di* represent the distances to the nearest atoms within the molecule and in adjacent molecules,^20,21^ respectively. The associated 2D fingerprint plots illustrate the *de vs di* distribution.^23^

### 2.2 Theoretical Analysis and Machine Learning Procedures

The analysis of the molecular and electronic structures of the compounds **QC-NO**_**2**_ and **QC-Cl** was carried out by Density Functional Theory (DFT),^24^ implemented in the Gaussian09 software package.^25^ Both compounds were initially optimized in the gas-phase using the M06–2*X*/6–311++G­(d,p) theory level.^26,27^ This level of theory presents satisfactory results, considering electronic correlation and noncovalent interactions.^26,28^ This optimization provides a description of the isolated geometry of the molecule, free of intermolecular interactions. A single-point energy calculation was also performed for the molecular geometry obtained through X-rays, without any additional geometric optimization (X-ray geometry). These calculations allow us to compare the electronic properties of the experimental molecular conformation with those obtained from the optimized geometry in the gas phase. This approach does not take into account the effects of long-range crystal packing, electrostatics, or the dispersion contributions inherent to the solid-state. From the wave function generated in these calculations, the frontier molecular orbitals (FMO) were calculated, which are the highest occupied molecular orbital (HOMO) and the lowest unoccupied molecular orbital (LUMO).^29^ From these parameters it is possible to determine some properties related to the chemical reactivity of the compounds, such as, the energy of the frontier molecular orbitals (E_HOMO_ and E_LUMO_), the energy difference between them (*E*_GAP_ = *E*_LUMO_ – *E*_HOMO_), the ionization potential (*I* ≅ – *E*_HOMO_), the electron affinity (*A* ≅ – *E*_LUMO_), the chemical potential (μ), the electronegativity (χ), the chemical hardness (η), and the electrophilicity index (ω), according to the equations:

1 $$\mu ={\left(\frac{\partial E}{\partial N}\right)}_{\upsilon }=-\frac{I+A}{2}=-\chi$$

2 $$\eta =\frac{1}{2}{\left(\frac{\partial^{2}E}{\partial N^{2}}\right)}_{\upsilon }=\frac{I-A}{2}$$

3 $$\omega =\frac{\mu^{2}}{2\eta }$$

in these equations, *E* is the energy of the system, *N* is the number of particles, *v* is the external potential. The molecular electrostatic potential (MEP) map was used to identify the nucleophilic and electrophilic regions^30,31^ of the compounds **QC-NO**_**2**_ and **QC-Cl**, and the electrostatic potentials *V*(**r**) values at the **r** point is defined by the equation:^32^

4 $$V\left(r\right)=\sum_{\alpha }\frac{Z_{\alpha }}{\left|r_{\alpha }-r\right|}-\int \frac{\rho \left(r^{\prime }\right)}{\left|r^{\prime }-r\right|}dr^{\prime }$$

where *Z*_α_ is the charge of nuclei α at point **r**_α_ and ρ­(**r**′) is the charge density at the point **r**′,^30^ and additionally the Fukui function to assist in predicting reactive sites was also calculated, using the equation:^33,34^

5 $$f\left(r\right)={\left[\frac{\partial \rho \left(r\right)}{\partial N}\right]}_{v}$$

where *N* is number of electrons in present system, the constant term *v* in the partial derivative is external potential. Fukui function can be calculated para nucleophilic, electrophilic, and radical attacks of the compounds occur, according to the equations:^35^

6 $$\begin{array}{ll} f^{+}\left(r\right)=\rho_{N+1}\left(r\right)-\rho_{N}\left(r\right)\approx \rho^{\mathrm{LUMO}}\left(r\right) & \left(n\mathrm{ucleophilic attack}\right) \end{array}$$

7 $$\begin{array}{ll} f^{-}\left(r\right)=\rho_{N}\left(r\right)-\rho_{N-1}\left(r\right)\approx \rho^{\mathrm{HOMO}}\left(r\right) & \left(e\mathrm{lectrophilic attack}\right) \end{array}$$

8 $$\begin{array}{ll} f^{0}\left(r\right)=\frac{f^{+}\left(r\right)+f^{-}\left(r\right)}{2}=\frac{\rho_{N+1}\left(r\right)-\rho_{N-1}\left(r\right)}{2}\approx \frac{\rho^{HOMO}\left(r\right)+\rho^{LUMO}\left(r\right)}{2} & \left(r\mathrm{adical attack}\right) \end{array}$$

Free-radical oxidation processes were modeled using a tool based on previously trained machine learning models by the *py*SiRC Platform^36^ available in http://www.pysirc.com.br/. This platform allows predictions of the reaction rate constant (*k*_OH_), which can be understood as the speed at which a compound reacts with hydroxyl radicals (^•^OH).^36–38^ These radicals (^•^OH) are highly reactive species, considered responsible for initiating and propagating oxidation in fuels, such as biodiesel.^2–4^ The (*k*_OH_) predictions were generated using the Molecular ACCess System keys - MACCS fingerprints^39,40^ in combination with a trained Extreme Gradient Boosting (XGBoost) model.^41^ MACCS fingerprints are 166-bit 2D structure fingerprints that are commonly used for the measure of molecular similarity,^42^ while the XGBoost algorithm employs parallelized gradient-boosted decision trees, offering high speed and accuracy in solving a wide range of data science problems. As a result, it has been extensively adopted in recent literature.^43–46^ To evaluate model performance, the following indices were calculated: the coefficient of determination (*R* ^2^), the Pearson correlation coefficient for predictions (*r* ^2^), the root-mean-square error (RMSE), and the external validation coefficient (*Q*_ext_^2^), according to the respective equations:

9 $$R^{2}=1-\frac{\sum_{i=1}^{n}{\left(y_{\exp}-y_{\mathrm{pred}}\right)}^{2}}{\sum_{i=1}^{n}{\left(y_{\exp}-{\mathrm{y̅}}_{\exp}\right)}^{2}}$$

10 $$r^{2}=\frac{\sum_{i=1}^{n}\left(y_{\exp}-{\mathrm{y̅}}_{\exp}\right).\left(y_{\mathrm{pred}}-{\mathrm{y̅}}_{\mathrm{pred}}\right)}{\sqrt{\sum_{i=1}^{n}{\left(y_{\exp}-{\mathrm{y̅}}_{\exp}\right)}^{2}\sqrt{\sum_{i=1}^{n}{\left(y_{\mathrm{pred}}-{\mathrm{y̅}}_{\mathrm{pred}}\right)}^{2}}}}$$

11 $$\mathrm{RMSE}=\sqrt{\frac{\sum_{i=1}^{n}{\left(y_{\exp}-y_{\mathrm{pred}}\right)}^{2}}{n}}$$

12 $$Q_{\mathrm{ext}}^{2}=1-\frac{\sum_{i=1}^{n}{\left(y_{\exp}-y_{\mathrm{pred}}\right)}^{2}}{\sum_{i=1}^{n}{\left(y_{\exp}-{\mathrm{y̅}}_{\exp}^{\mathrm{tr}}\right)}^{2}}$$

Where *y̅*_exp_^*tr*^ is the average value of the dependent variable for the training set, *y*_exp_ are the experimental values, *y*_pred_ are the predicted values, while *y̅*_exp_ and *y̅*_pred_ and average of the experimental and predicted values of the dependent variable, respectively.^36^ According to the *py*SiRC developers, the trained model exhibited excellent agreement with the training data (*R* ^2^ > 0.937) with strong predictive performance on the test set, with external validation coefficients (*R*_ext_^2^ = *Q*_ext_^2^) ranging from 0.707 to 0.823.^36^ The platform also calculates the similarity between the target compounds and the training set (Applicability domain - AD%), to support the reliability of the predictive model.^36^ The AD verifies whether a query compound is represented within the chemical space of the training set, allowing the model to provide trustworthy predictions. Structural similarity between the query molecule and each compound from the training data set was quantified using the Tanimoto coefficient:

13 $$T_{c}\left(A,B\right)=\frac{c}{a+b-c}$$

where *a* and *b* correspond to the number of features present in compounds *A* and *B*, respectively, and *c* is the number of shared features between them. The developers of *py*SiRC validated their predictions of hydroxyl radical scavenging rate constants (*k*_OH_) by comparing them with experimental values widely cataloged in the literature, including Supporting data from Zhong et al. al.,^47^ Borhani et al.,^48^ Ortiz et al.,^49^ Luo et al.,^37^ Wojnárovits & Takács,^50^ as well as the databases IscoKin^51^ e NIST.^52^ The complete data set used by the developers for validation is available in the Supporting Information.^36^ The developers reported that the predictions showed good agreement with experimental values, even for molecules with intermediate applicability domains (60–75%), a range equivalent to that observed in our study.

The *k*_OH_ parameters were calculated for the compounds under study (**QC-NO**_**2**_ and **QC-Cl**) and compared with the *k*_OH_ indices of diesel fuels and representative biodiesel (BD) compounds, including methyl 9-octadecenoate (BD M9O) and methyl palmitate (BD MPA),^53^ and with some commercial additives (butyl hydroxyanisole (BHA), *tert*-butylhydroquinone (TBHQ), butylhydroxytoluene (BHT), gallic acid (GA), pyrogallol (PY) and propyl gallate (PG)).^7^ The analyzed molecules can be set on the platform in ASCII format, with the SMILES identifiers of the compounds. The *k*_OH_ prediction is interesting since an antioxidant can act as a scavenger or shield that reacts preferentially with free radicals (such as ^•^OH), preventing these radicals from attacking the fuel molecules (biodiesel or diesel), which would cause degradation (oxidation).^36–38^ Therefore, the higher the *k*_OH_ constant of the antioxidant, theoretically the faster it would react with hydroxyl radicals, consuming them before these radicals cause damage to the fuel, which is the expected role of a fuel additive compound.^5,6^ The antioxidant activity of chalcone derivatives against hydroxyl radicals generally proceeds through Hydrogen Atom Transfer (HAT)^54,55^ or Single Electron Transfer (SET) mechanisms,^55^ as previously described for structurally related aromatic and conjugated systems.^56–58^ These pathways involve either direct H-abstraction from activated sites or electron transfer followed by proton release, both of which correlate with the electronic descriptors evaluated in this work.

## 3 Results and Discussion

### 3.1 Solid-State Description

The crystal structures of **QC-NO**_**2**_ and **QC-Cl** molecules were determined by single-crystal X-ray diffraction analysis. The molecules are structurally similar, a quinolinone-chalcone core and four aromatic rings, along with a sulfonamide group and a nitro group at the *para* position of aromatic ring 3. The only structural difference between the two compounds lies in the substitution at the *para* position of the aromatic ring 2. **QC-Cl** contains a chlorine atom, whereas **QC-NO**_**2**_ features a nitro group at this position (Figure 1). Both molecules crystallize in the triclinic P1̅, with similar unit cell parameters, as expected for isomorphic compounds (Table S1). Both structures contain solvent molecules; however, since these solvents did not participate in significant intermolecular interactions within the supramolecular arrangement, they were omitted from subsequent structural and theoretical analyses.

**Figure 1:**
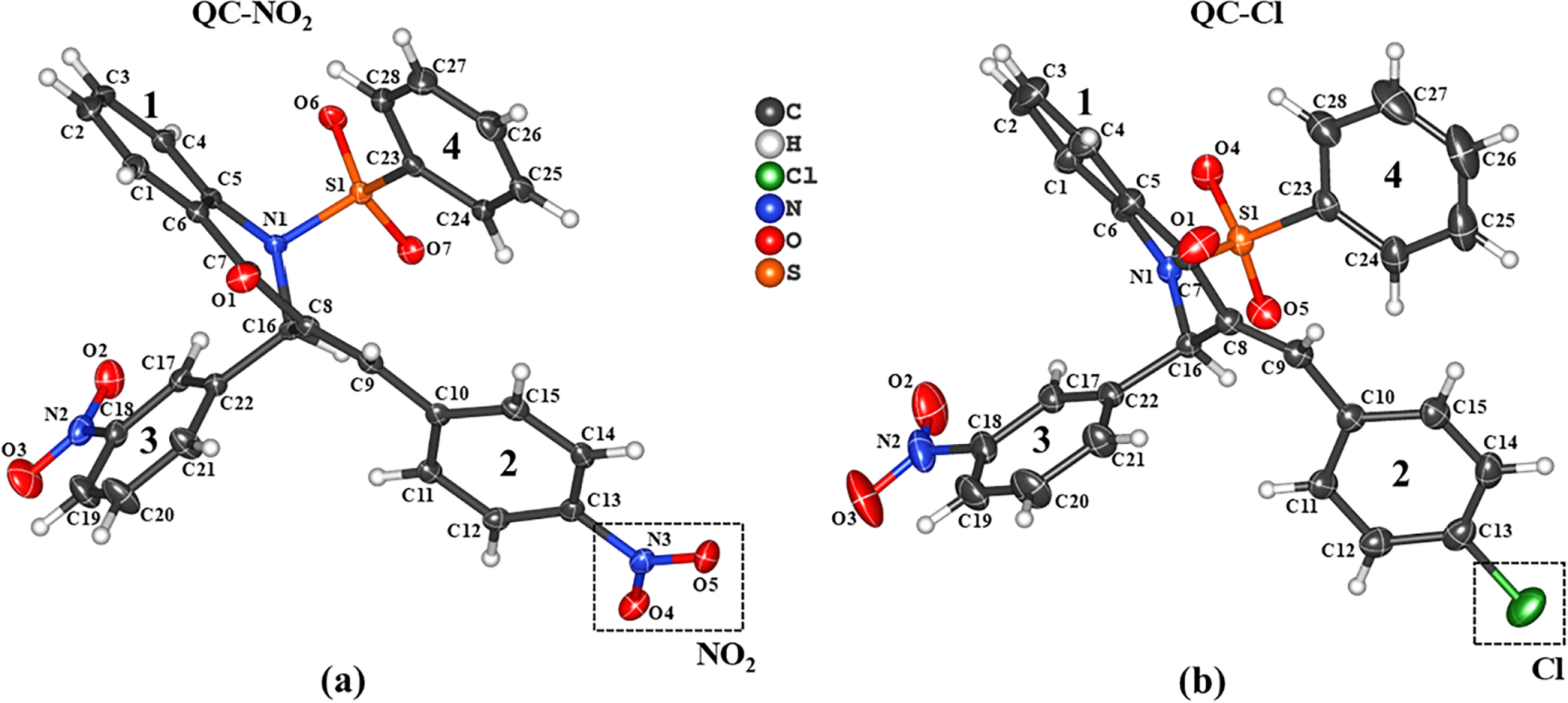
ORTEP representation of **QC-NO**_**2**_ (a) and **QC-Cl** (b) are shown with displacement ellipsoids drawn at the 30% probability level; hydrogen atoms are depicted as spheres of arbitrary radius for clarity.

The molecular conformation of a compound is associated with its potential biological activity, as well as to the presence of substituent groups.^11,59^ Studies indicate that in chalcones, molecular conformation, including planarity, affects electron and hydrogen atom transfer mechanisms, which play a crucial role to antioxidant activity.^8,12^ The substitution of the nitro group in **QC-NO**_**2**_ introduces different intermolecular interactions and results in a distinct molecular packing arrangement (Figure 2a). However, structural overlay of the substituted ring region in QC-NO_2_ and QC-Cl yields an RMSD of only 0.0421 (Figure 2b), indicating that the nitro and chloride substituents do not significantly change the molecular conformation. This suggests that the differences observed in molecular packing are primarily driven by the distinct electronic nature and intermolecular interaction profiles of the substituents rather than by conformational divergence.

**Figure 2:**
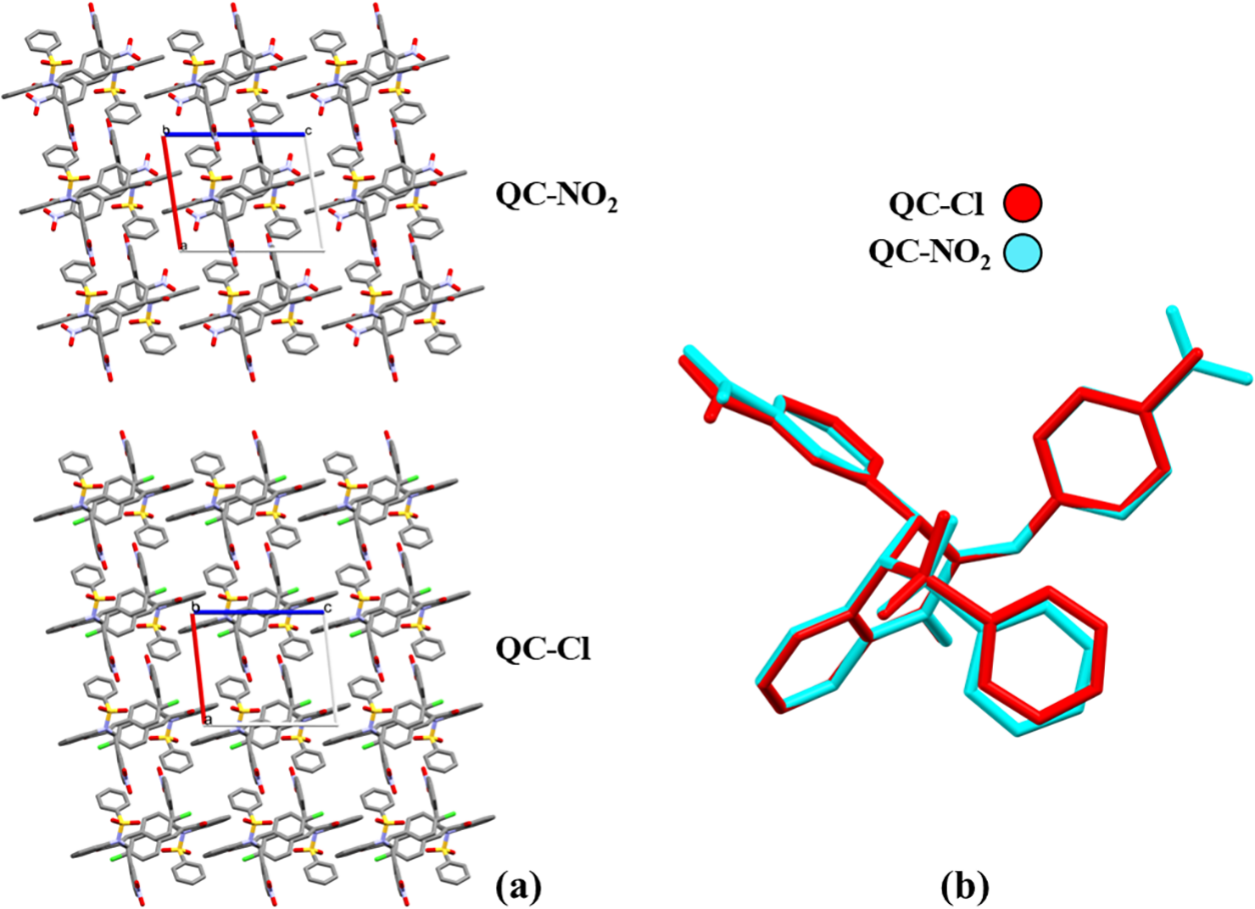
Molecular packing in the unit cell (a) and overlaps between compounds **QC-NO**_**2**_ and **QC-Cl** (b).

The crystal structure of **QC-NO**_**2**_ exhibits a supramolecular arrangement stabilized by several nonclassical hydrogen bonds (C–H···O) (Figure 3a). The interactions C15–H15···O3 and C26–H26···O2 contribute along the [100] and [010] axes. These contacts feature H···A distances of 2.680(3) Å and 2.699(5) Å, and D–H···A angles of 167.35(5)° and 127.56(3)°, respectively (Table 1). A more intricate network is observed along the [001] axis, where five distinct C–H···O contacts C28–H28···O4, C27–H27···O5, C12–H12···O6, C16–H16···O7, and C3–H3···O6 are present. These interactions exhibit H···A distances ranging from 2.440(4) Å to 2.643(4) Å and angles between 127.59(4)° and 154.75(3)°, indicating a range of interaction strengths along the [001] axis. Overall, the supramolecular arrangement of **QC-NO**_**2**_ is driven by a network of weak yet directional C–H···O hydrogen bonds. In addition, the crystal structure of **QC-Cl** also exhibits a supramolecular arrangement stabilized by C–H···O hydrogen bonds (Figure 3b). A notable C–H···O interaction extends simultaneously along both the [100] and [010] directions, involving a C–H group interacting with the oxygen atom O5CA. This contact, characterized by a H···A, distance of 2.610(3) Å and a D–H···A angle of 139.78(5)°, contributes to establishing the lateral arrangement of the molecules within the unit cell. Along the [001] axis, three additional directional C–H···O interactions play a central role in constructing the vertical coherence of the crystal. These include C5BA–H5BA···O2AA, characterized by an H···A distance of 2.467(4) Å and a nearly linear angle of 166.59(4)°, suggesting a relatively strong supramolecular interaction. The C4BA–H4BA···O2AA (2.604(3) Å, 147.58(4)°) and C3AA–H3AA···O1AA (2.671(3) Å, 127.69(3)°) interactions further contribute to the vertical assembly of the molecular structure.

**Figure 3:**
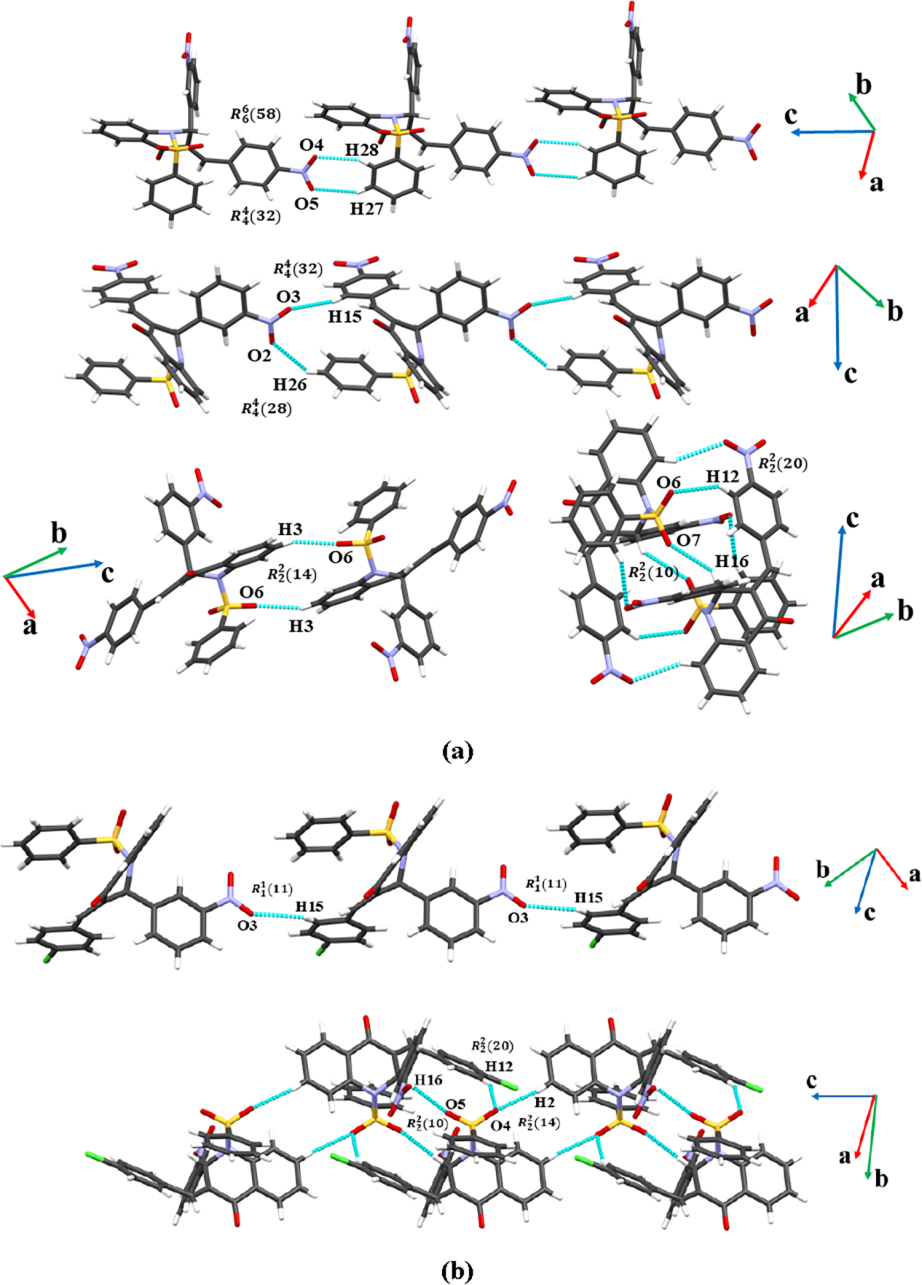
Representations for **QC-NO**_**2**_ (a) and **QC-Cl** (b) interactions.

**Table 1:** Main Supramolecular Interactions Observed in the **QC-NO**_**2**_ and **QC-Cl** Compounds

	**interactions**	**d(D–H) Å**	**d(H···A) Å**	**d(D–A) Å**	**(D–H–A) (°)**	**symmetry code**
**QC-NO**_**2**_	C27–H27···O5	0.950	2.643(4)	3.437(3)	141.36(4)	*x*, *y*, -1 + *z*
	C28–H28···O4	0.950	2.511(4)	3.379(3)	151.68(3)	*x*, *y*, 1 + *z*
	C15–H15···O3	0.950	2.680(3)	3.614(4)	167.35(5)	–1 + *x*, 1 + *y*, *z*
	C26–H26···O2	0.950	2.699(5)	3.363(3)	127.56(3)	–1 + *x*, 1 + *y*, z
	C12–H12···O6	0.950	2.451(3)	3.245(4)	140.53(5)	1–*x*, 2–*y*, 1–*z*
	C16–H16···O7	1.000	2.578(4)	2.896(3)	127.59(4)	1–*x*, 2–*y*, 1–*z*
	C3–H3···O6	0.950	2.440(4)	3.325(4)	154.75(3)	1–*x*, 2–*y*, 2–*z*
**QC-Cl**	C15–H15···O3	0.930	2.610(3)	3.374(5)	139.78(5)	1 + *x*, −1 + *y*, *z*
	C2–H2···O4	0.930	2.467(4)	3.378(3)	166.59(4)	2–*x*, −*y*, 1–*z*
	C12–H12···O4	0.930	2.604(3)	3.425(5)	147.58(4)	2–*x*, −*y*, 1–*z*
	C16–H16···O5	0.980	2.671(3)	3.361(2)	127.69(3)	2–*x*, −*y*, 1–*z*

The *d*_norm_ HS confirmed the interactions previously identified from geometric parameters for both molecules. As shown in Figure 4, the highlighted regions on the surfaces support this interpretation: white areas indicate contacts close to the van der Waals separation, whereas red regions correspond to contacts shorter than the sum of the van der Waals radii. The intermolecular contacts present in both molecules were quantitatively classified by type using bidimensional (2D) fingerprint plots derived from HS (Figure 5). These 2D fingerprints exhibit pseudomirrored spikes at 1.0 (*d*_*e*_, *d*_*i*_) – 1.2­(*d*_*e*_, *d*_*i*_), indicating the presence of H···H interactions, which account for 23.0% and 29.6% of all contacts in **QC-NO**_**2**_ and **QC-Cl**, respectively. Peaks around 1.2–1.4 Å (*d*_*e*_ and *d*_*i*_) suggest the presence of C–H···O interactions, contributing 37.1% (**QC-NO**_**2**_) and 29.5% (**QC-Cl**). Contacts involving π-systems, such as C–H···π and π···π interactions, are also present, with contributions of 17.3 and 5.3% for **QC-NO**_**2**_, and 17.6 and 4.6% for **QC-Cl**, respectively. Altogether, these interactions represent approximately 80% of all intermolecular contacts, indicating that both molecules are predominantly stabilized by weak C–H···O hydrogen bonds.

**Figure 4:**
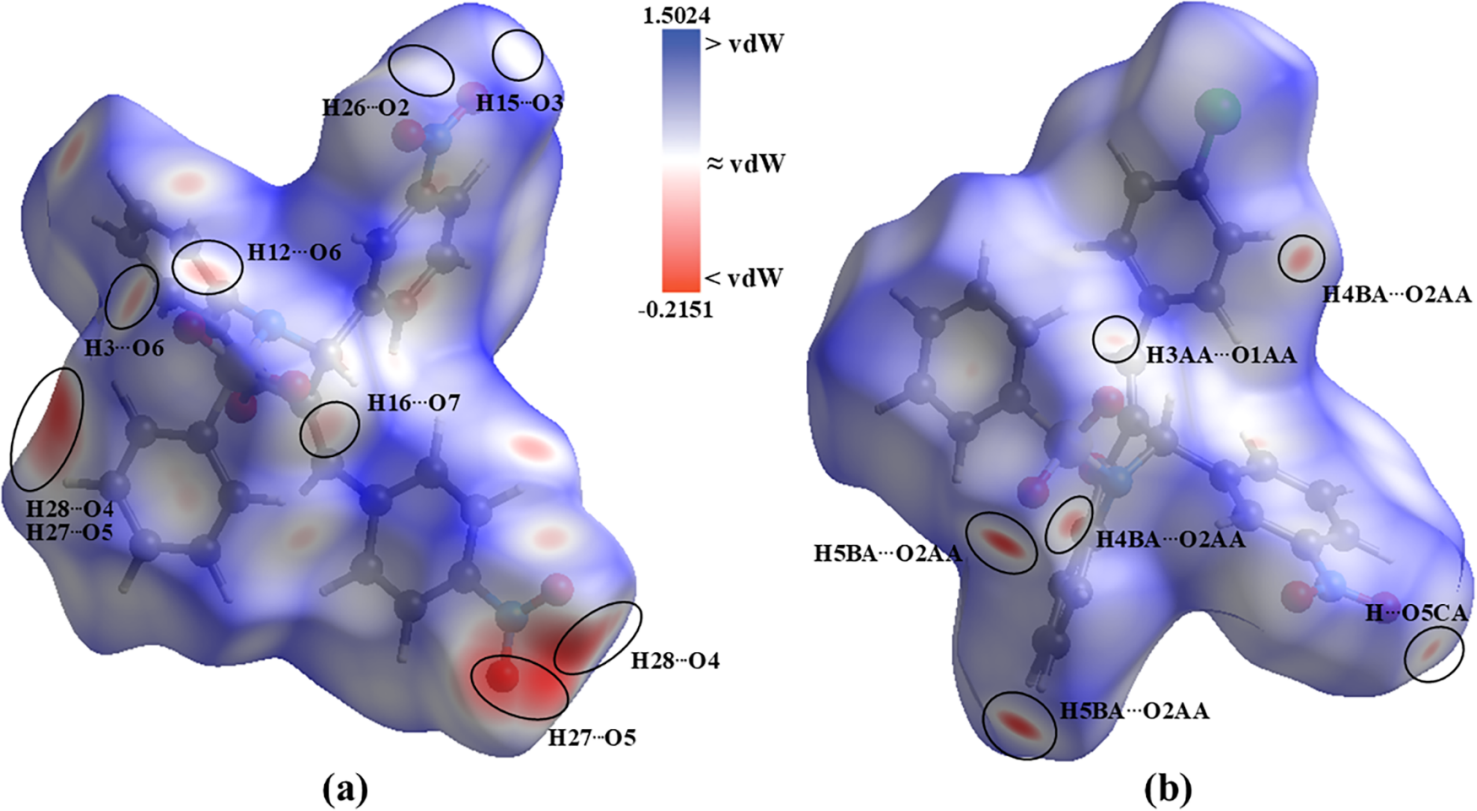
Hirshfeld surface (*d*_norm_) indicates the interactions for **QC-NO**_**2**_ (a) and **QC-Cl** (b).

**Figure 5:**
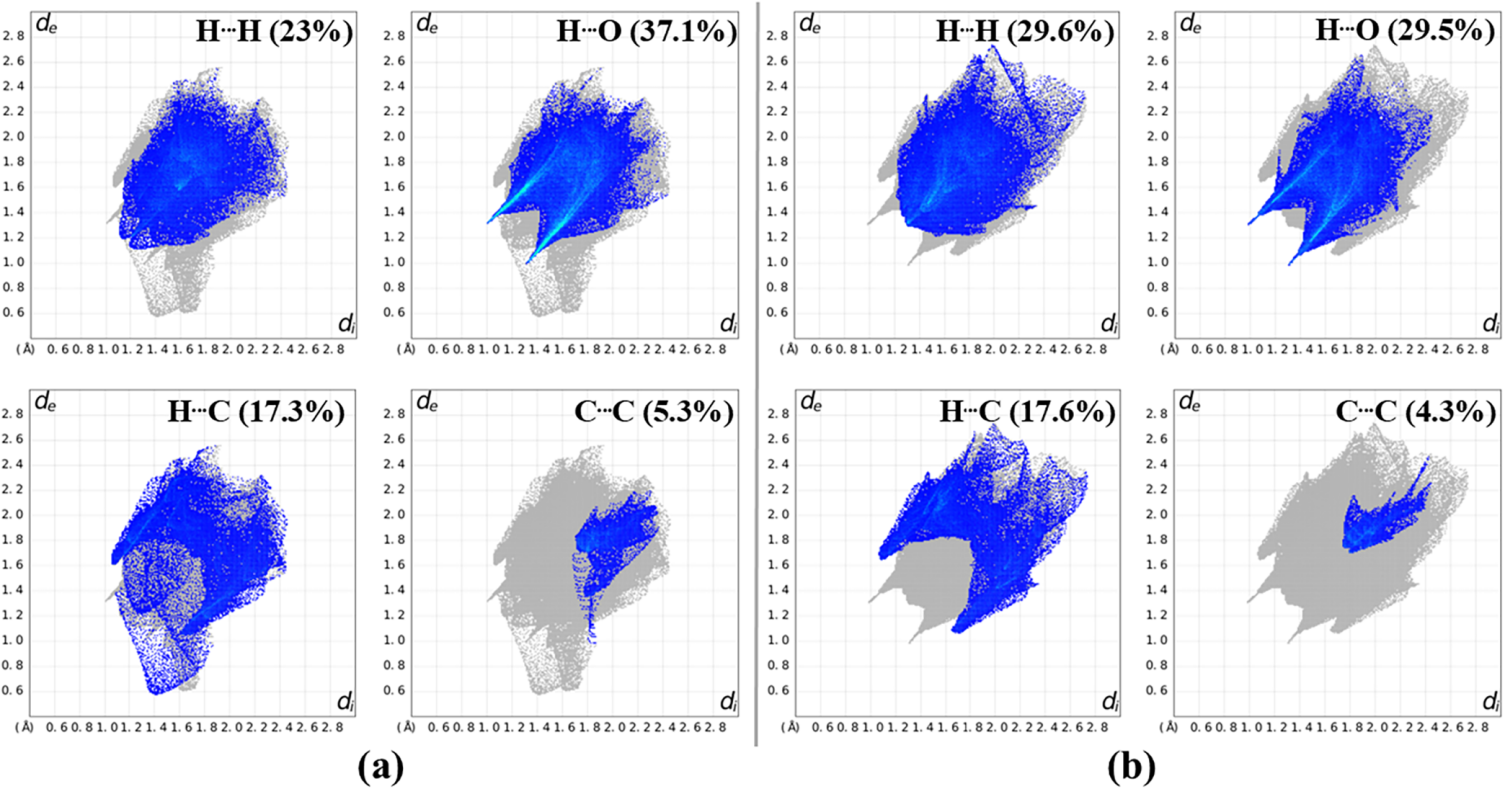
2D fingerprint plots showing the percentage contributions of the different contact types for **QC-NO**_**2**_ (a) and **QC-Cl** (b).

The solid-state analysis presented provides the structural foundation for the subsequent discussions on electronic properties and reactivity. The intermolecular C–H···O and C–H···π interactions, identified by geometric parameters and electron density, as well as the molecular packing patterns observed in the crystal structures, influence the electron distribution, molecular stability, and potential radical-trapping sites explored. These supramolecular characteristics help explain how differences in frontier orbital energies, global reactivity descriptors, and Fukui indices connect the solid-state conformation to the antioxidant activity proposed for **QC-NO**_**2**_ and **QC-Cl**.

### 3.2 Molecular Modeling Analysis

The analysis of chemical reactivity descriptors (Table 2) and molecular orbitals (Figure 6) reveals important differences between the compounds **QC-NO**_**2**_ and **QC-Cl**, both in the gas phase and in the X-ray geometry. According to the values obtained, the compounds present greater electronic stability in the X-ray geometry, evidenced by the increase in *E*_GAP_ values, lower levels of *E*_HOMO_ and *E*_LUMO_, and an increase in ionization energy (*I*) and electronic affinity (*A*).^60,61^ Comparatively, **QC-Cl** showed the highest *E*_GAP_ in both phases (597.75 and 599.87 kJ/mol), indicating a more electronically stable and less reactive structure. Nevertheless, **QC-NO**_**2**_ showed a higher electrophilicity index (ω), suggesting a greater tendency to accept electron density, which under the conditions evaluated, reflects a more pronounced electrophilic character. **QC-Cl**, with higher *E*_HOMO_ values, proved to be a better electron donor (greater nucleophilicity), while **QC-NO**_**2**_ stood out as the best acceptor.^62,63^ These results indicate that the nitro substituent contributes to a greater electronic reactivity of the system, while the chlorine substituent promotes greater stability and chemical hardness. The reactivity descriptors indicate that **QC-Cl**, due to its higher E_HOMO_ values, has a greater tendency to donate electrons, thus behaving as a potential nucleophile in electron exchange interactions. In contrast, **QC-NO**_**2**_, with a lower *E*_LUMO_ and higher electrophilicity index (ω), behaves as a better electron acceptor, favoring its performance as an electrophile in redox processes. These results are consistent with the electronic nature of the substituents: the nitro group (NO_2_) acts as a strong electron-withdrawing group, enhancing the molecule’s ability to accept electrons, while chlorine (Cl), being weakly electron-donating through resonance and inductive effects, contributes to electron stabilization and donation. Such substituent effects can directly influence the antioxidant activity and the reactive profile of these compounds.

**Figure 6:**
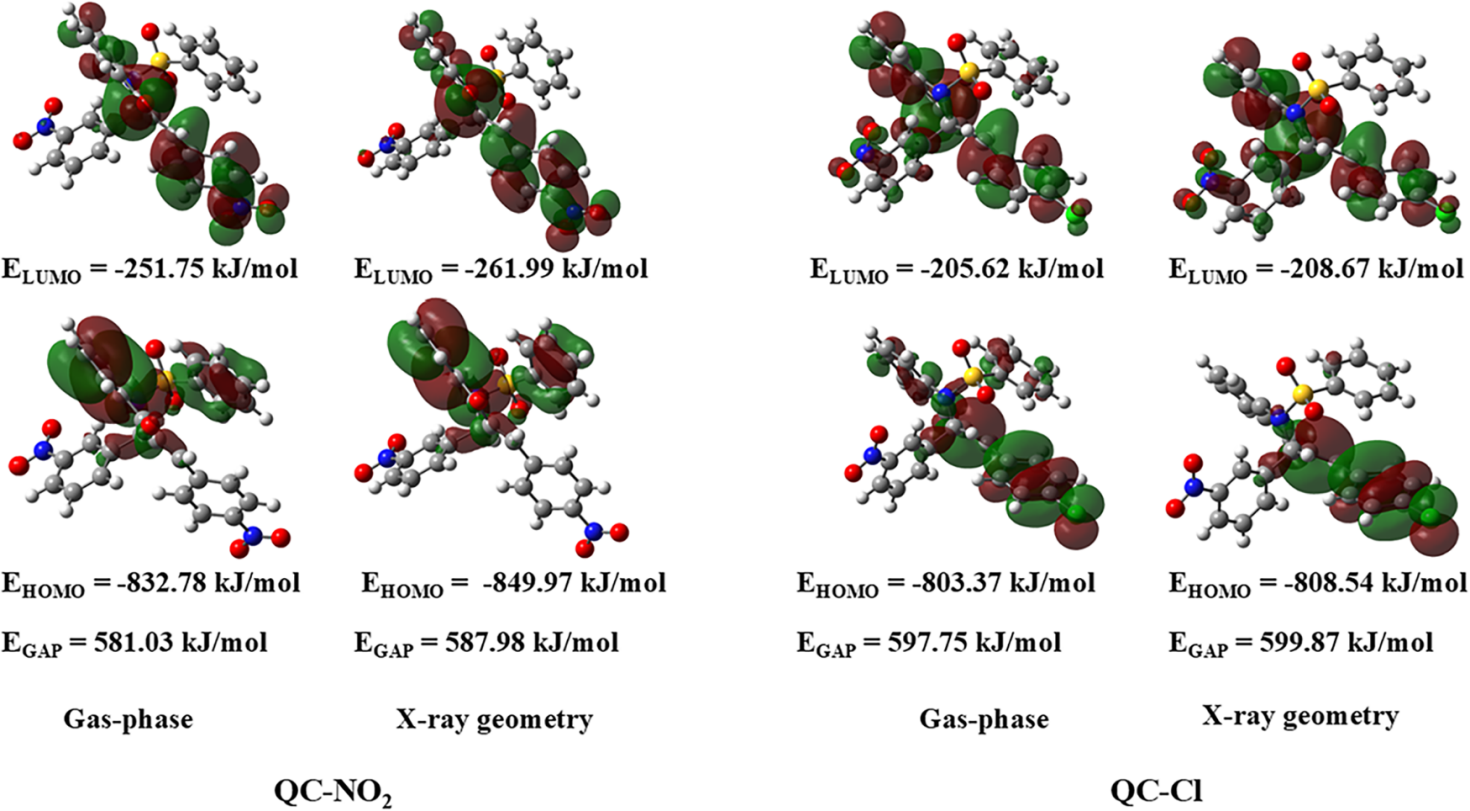
FMO orbitals for **QC-NO**_**2**_ and **QC-Cl** in the gas-phase and X-ray geometry.

**Table 2:** Chemical Reactivity Descriptors of the **QC-NO**_**2**_ and **QC-Cl** Compounds^a^

**descriptor**	**QC-NO**_**2**_ **(gas-phase)**	**QC-NO**_**2**_ **(X-ray geometry)**	**QC-Cl (gas-phase)**	**QC-Cl (X-ray geometry)**
HOMO energy (*E*_HOMO_)	–832.78	–849.97	–803.37	–808.54
LUMO energy (*E*_LUMO_)	–251.75	–261.99	–205.62	–208.67
energy gap (*E*_GAP_)	581.03	587.98	597.75	599.87
ionization energy (*I*)	832.78	849.97	803.37	808.54
electronic affinity (*A*)	251.75	261.99	205.62	208.67
chemical potential (μ)	–542.26	–555.98	–504.49	–508.60
electronegativity (χ)	542.26	555.98	504.49	508.60
chemical hardness (η)	290.51	293.99	298.87	299.87
electrophilicity index (ω)	506.08	525.72	425.78	431.31

a All units are in kJ/mol.

The presence of the chlorine substituent in **QC-Cl** increases the *V*(***r***) value in the nitro and sulfonamide groups when compared to the same regions of **QC-NO**_**2**_ (Figure 7). The regions of negative potential (represented in red) are mainly concentrated around electronegative atoms, such as oxygen and nitrogen, indicating nucleophilic regions prone to act as hydrogen bond acceptors and to interact with electrophilic species, such as free radicals. On the other hand, the regions in blue (areas of positive potential) are associated with hydrogen bonded to electronegative groups, indicating hydrogen-bond donor sites. The asymmetric distribution of these potentials in **QC-NO**_**2**_ and **QC-Cl** suggests a significant polarity, which may favor the formation of supramolecular interactions that can influence properties such as solubility, stability, and antioxidant potential of the molecule.

**Figure 7:**
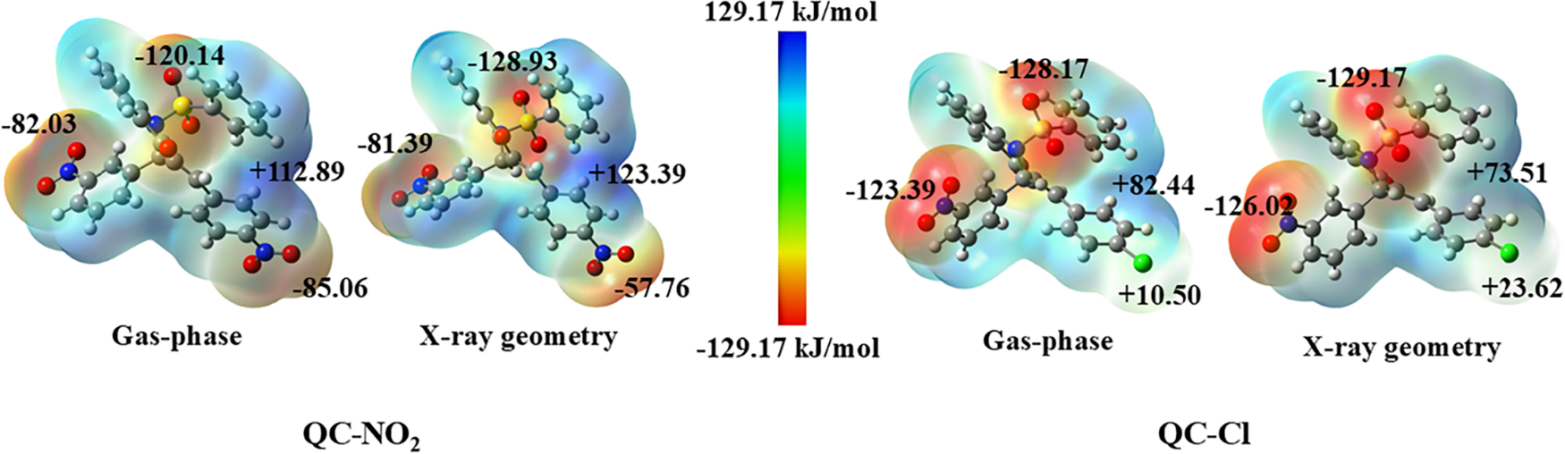
MEP map surfaces for **QC-NO**_**2**_ and **QC-Cl** in the gas-phase and X-ray geometry.

The Fukui function was employed to predict the local reactivity of **QC-NO**_**2**_ and **QC-Cl**, highlighting the regions most susceptible to nucleophilic [*f* ^+^(***r***)], electrophilic [*f* ^–^(***r***)], and radical attacks [*f* ^0^(***r***)] (Figure 8). The Fukui function profiles for **QC-NO**_**2**_ showed close agreement between the X-ray geometry and gas-phase calculations, a trend also observed for **QC-Cl**. For **QC-NO**_**2**_, the [*f* ^+^(***r***)] function revealed that the regions near the oxygen atoms of the sulfonamide group, as well as the nitro substituent attached to aromatic ring 2, are the most favorable sites for nucleophilic attack. In contrast, the [*f* ^–^(***r***)] function indicated that the central nitrogen atom is the most prone to electrophilic attack. Similarly, for **QC-Cl**, the [*f* ^+^(***r***)] function indicated the central oxygen atom as the preferential site for nucleophilic attack, whereas the [*f* ^–^(***r***)] function again identified the central nitrogen as the most electrophilic site. In both molecules, the [*f* ^0^(***r***)] function overlapped with the sites predicted by [*f* ^+^(***r***)] and [*f* ^–^(***r***)], confirming that the susceptibility to radical attack is directly associated with regions that can either accept or donate electron density. Note that the nucleophilic and electrophilic reactive sites are similar for **QC-NO**_**2**_ and **QC-Cl**. However, the *nitro-chloride* substitution at the *para* position of aromatic ring 2 enhances the susceptibility of **QC-NO**_**2**_ to nucleophilic attack, reflecting the strong electron-withdrawing effect of the nitro group, reflecting the strong electron-withdrawing effect of the nitro group. These findings highlight the key role of substituent effects in local reactivity and support the interpretation that electron withdrawal by -NO_2_ increases the electrophilic character of adjacent sites.

**Figure 8:**
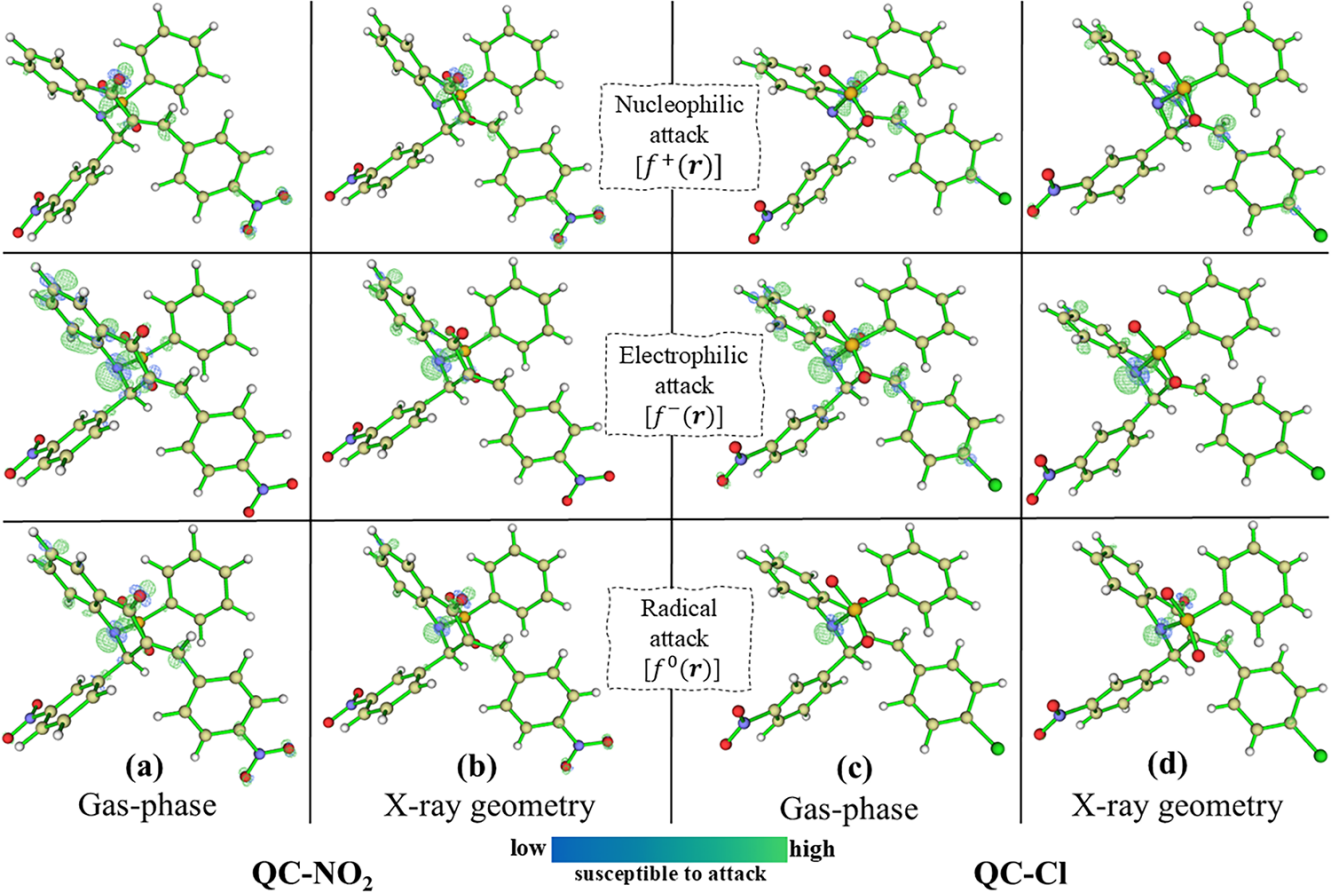
Isosurfaces of the **QC-NO**_**2**_ Gas-phase (a), **QC-NO**_**2**_ X-ray geometry (b), **QC-Cl** Gas-phase (c) and **QC-Cl** X-ray geometry (d), showing the nucleophilic *f* ^+^(***r***), electrophilic *f* ^–^(***r***), and radical attack regions *f* ^0^(***r***), calculated at a value of 0.007. Color scale: blue indicates regions with low susceptibility to chemical attack, while green indicates regions with higher susceptibility to attack.

The predicted values of the reaction constant with the hydroxyl radical (*k*_OH_) using MACCS descriptors indicate that the compounds **QC-NO**_**2**_ and **QC-Cl** have significant potential as antioxidant aditives in biodiesel blends (Table 3). The **QC-NO**_**2**_ compound presented a *k*_OH_ of 6.09 × 10^9^ M^–1^·s^–1^, a value higher than some commercial biodiesels (BD) evaluated, such as BD M9O (5.94 × 10^9^ M^–1^·s^–1^) and BD MPA (4.98 × 10^9^ M^–1^·s^–1^). These results suggest that **QC-NO**_**2**_ may be a promising candidate for mitigating oxidation promoted by hydroxyl radicals, supporting its potential as an antioxidant additive for biodiesel. The applicability domain (AD%) for **QC-NO**_**2**_ was 64.20%, which demonstrates moderate reliability in the prediction, indicating that the statistical model employing MACCS recognizes **QC-NO**_**2**_ as similar to compounds in the training set.^36^ Note that molecules structurally distant from the training set or with low AD% values may present greater uncertainties in their predicted rate constants,^36^ the AD% for **QC-NO**_**2**_ is moderate, and future studies should include experimental kinetic measurements to provide a direct reference and further validate the predicted *k*_*OH*_ values. Additionally, the compound **QC-Cl** exhibited a lower *k*_OH_ of 2.51 × 10^9^ M^–1^·s^–1^, but still significant, associated with an AD% of 61.18%, similar to **QC-NO**_**2**_. This result indicates moderate antioxidant potential, which may be useful in specific biodiesel formulations or in combination with other antioxidant additives. When comparing the compounds **QC-NO**_**2**_ and **QC-Cl** with some commercial additives, such as TBHQ (7.63 × 10^9^ M^–1^·s^–1^), BHT (4.34 × 10^9^ M^–1^·s^–1^), BHA (4.31 × 10^9^ M^–1^·s^–1^), GA (1.48 × 10^9^ M^–1^·s^–1^), PG (1.22 × 10^10^ M^–1^·s^–1^) and PY (1.02 × 10^10^ M^–1^·s^–1^), it is noted that **QC-NO**_**2**_ is favorably positioned in terms of potential antioxidant activity when we compare *k*_OH_ values, which are close to or higher than some industrial and commercial standards. Therefore, the results suggest that both compounds, especially **QC-NO**_**2**_, are promising candidates for antioxidant additives in biodiesel, with the potential to compete with established commercial antioxidants. Future investigations, including experimental assays and evaluation of compatibility with biodiesel blends, are needed to validate these computational findings.

**Table 3:** Reaction Rate (*k*_OH_) for **QC-NO**_**2**_ and **QC-Cl** ^a^ ^b^

	**reaction rate coefficient (** ***k***_**OH**_ **) (**M^**–1**^ **s** ^**–1**^ **)**	
**molecule**	AD (%)	MACCS
QC-NO_2_	64.20	6.09 × 10^9^
QC-Cl	61.18	2.51 × 10^9^
Diesel	76.92	1.14 × 10^10^
BD M9O	85	5.94 × 10^9^
BD MPA	89.47	4.98 × 10^9^
TBHQ	100	7.63 × 10^9^
BHT	100	4.34 × 10^9^
BHA	77.78	4.31 × 10^9^
GA	100	1.48 × 10^9^
PG	100	1.22 × 10^10^
PY	100	1.02 × 10^10^

a The *k*_OH_ also are presents for other commercial additives, and for Diesel and biodiesel (BD) by their majority compound, respectively. AD is the % of similarity within the applicability domain.

b Biodiesel methyl 9-octadecenoate (BD M9O); Biodiesel methyl palmitate (BD MPA); Tert-butylhydroquinone (TBHQ); Butylhydroxytoluene (BHT); Butylhydroxyanisole (BHA); Gallic acid (GA); Propyl gallate (PG); Pyrogallol (PY).

## 4 Conclusions

In this study, we report the analysis of two quinolinone-chalcone derivatives, **QC-NO**_**2**_ and **QC-Cl**, to investigate how their *nitro* and *chloro* substituents affect their molecular properties and antioxidant potential. Crystallographic analyses showed that both compounds have similar conformations and supramolecular arrangements stabilized by C–H···O interactions. However, the substituents directly influenced molecular packing and electronic characteristics: **QC-NO**_**2**_ proved to be more electrophilic, while **QC-Cl** showed greater electronic stability.

DFT simulations confirmed that **QC-NO**_**2**_ acts as a stronger electron acceptor, while **QC-Cl** is a more effective electron donor, which was corroborated by electrostatic potential maps and Fukui function predictions of local reactivity. Estimates of hydroxyl radical reactivity (*k*_OH_) obtained from a tool based on previously trained machine learning models indicate that **QC-NO**_**2**_ has an activity comparable to, or higher than, certain commercial additives, **QC-Cl** shows moderate activity, yet remains within the range of applicability for potential additives. In summary, our results highlight the importance of *nitro-chloro* substituent effects in chalcone hybrids: **QC-NO**_**2**_ presents antioxidant potential for biodiesel applications, and **QC-Cl** can be used in complementary formulations.

## Supplementary Material



## Notes

Conditions for the synthesis (Scheme S1); ^1^H NMR, ^13^C NMR, HRMS and FTIR spectra for QC-NO_2_ and QC-Cl (Figures S1–S8); and Crystallographic data for QC-NO_2_ and QC-Cl (Table S1) (PDF)
