# Supplementary material for: The Nitro-Chloro Substitution on Two Quinolinone-Chalcones: From Molecular Modeling to Antioxidant Potential
Source: ACS Omega. 2026 Feb 3;11(6):10269–79. doi: 10.1021/acsomega.5c11439 (PMC12917819; doi:10.1021/acsomega.5c11439)
Supplement: Supplementary file 1 [file ao5c11439_si_001.pdf]

Supporting Information

The *nitro-chloro* substitution on two quinolinone-chalcones: from molecular modeling to antioxidant potential

Renata Layse G. de Paula[a]*, Vitor S. Duarte[a], Giulio D. C. D'Oliveira[c], Mirian R. C. de Castro[c], Caridad N. Pérez[c], Jean M. F. Custódio[a], Allen G. Oliver[b] and Hamilton B. Napolitano[a,b]*

[a] Universidade Estadual de Goiás, Grupo de Química Teórica e Estrutural, 75132-903, Anápolis, GO, Brazil.
[b] University of Notre Dame, Department of Chemistry and Biochemistry, 46556, Notre Dame, IN, USA.
[c] Universidade Federal de Goiás, Instituto de Química, 74690-900, Goiânia, GO, Brazil.

*renata.fisicaueg@gmail.com
*hbnapolitano@gmail.com

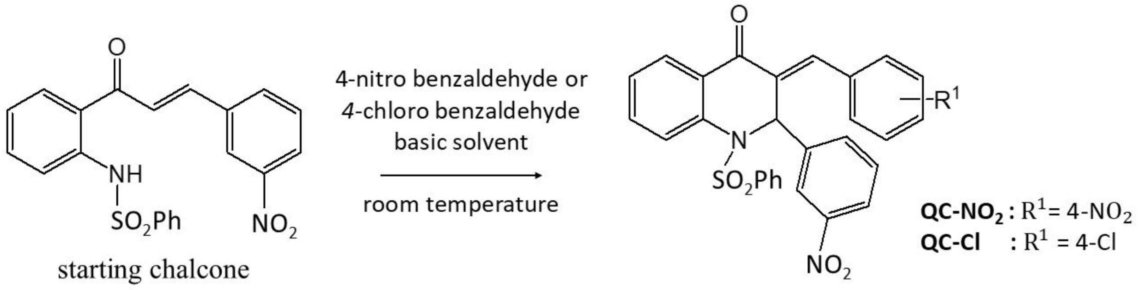

Scheme S1. Conditions for the synthesis of *(E)-3-(4-nitrobenzylidene)-2-(3-nitrophenyl)-2,3-dihydro-1-(phenylsulfonyl)-quinolin-4(1H)-one* (**QC-NO$_2$**) and *(E)-3-(4-chlorobenzylidene)-2-(3-nitrophenyl)-2,3-dihydro-1-(phenylsulfonyl)-quinolin-4(1H)-one* (**QC-Cl**).

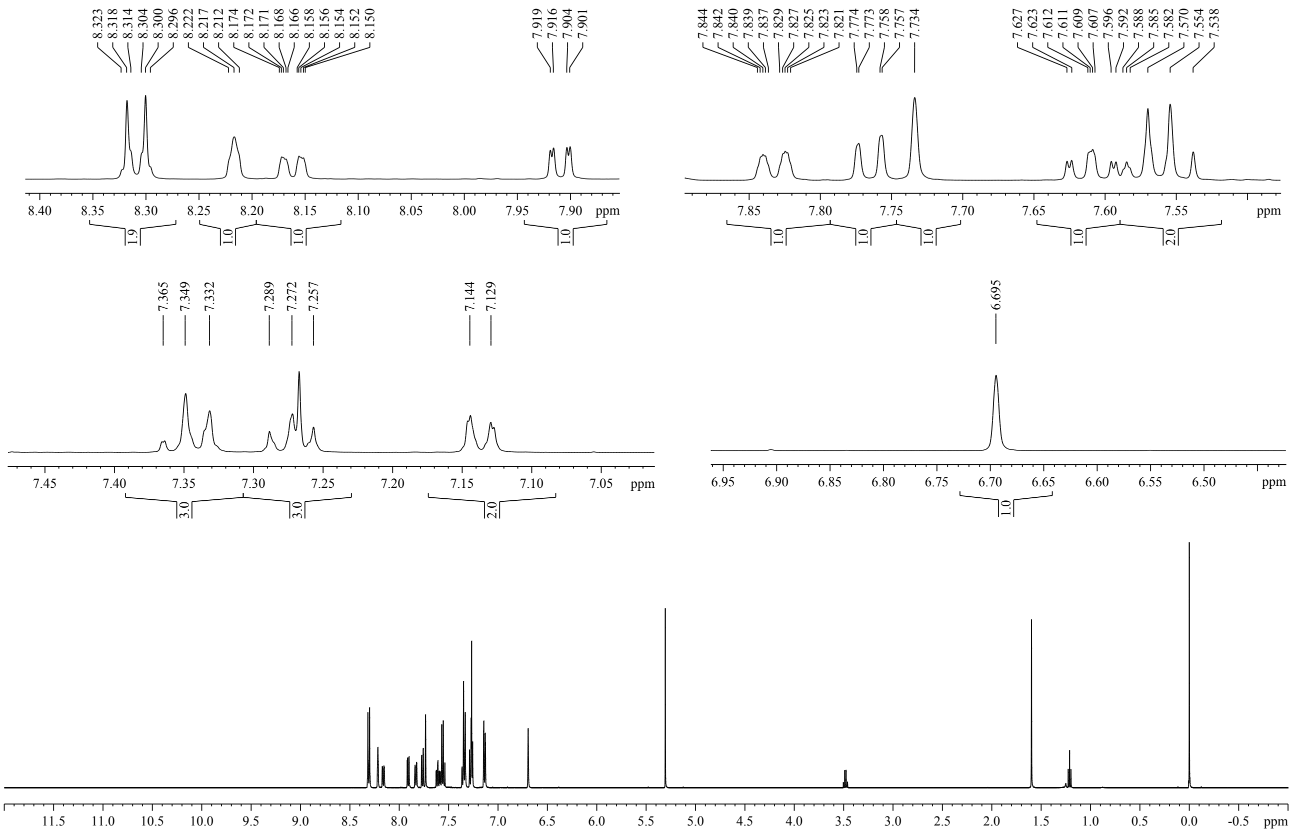

Figure S1. ^{1}H NMR spectrum (500 MHz, $CDCl_3$) of compound **QC-NO$_2$**.

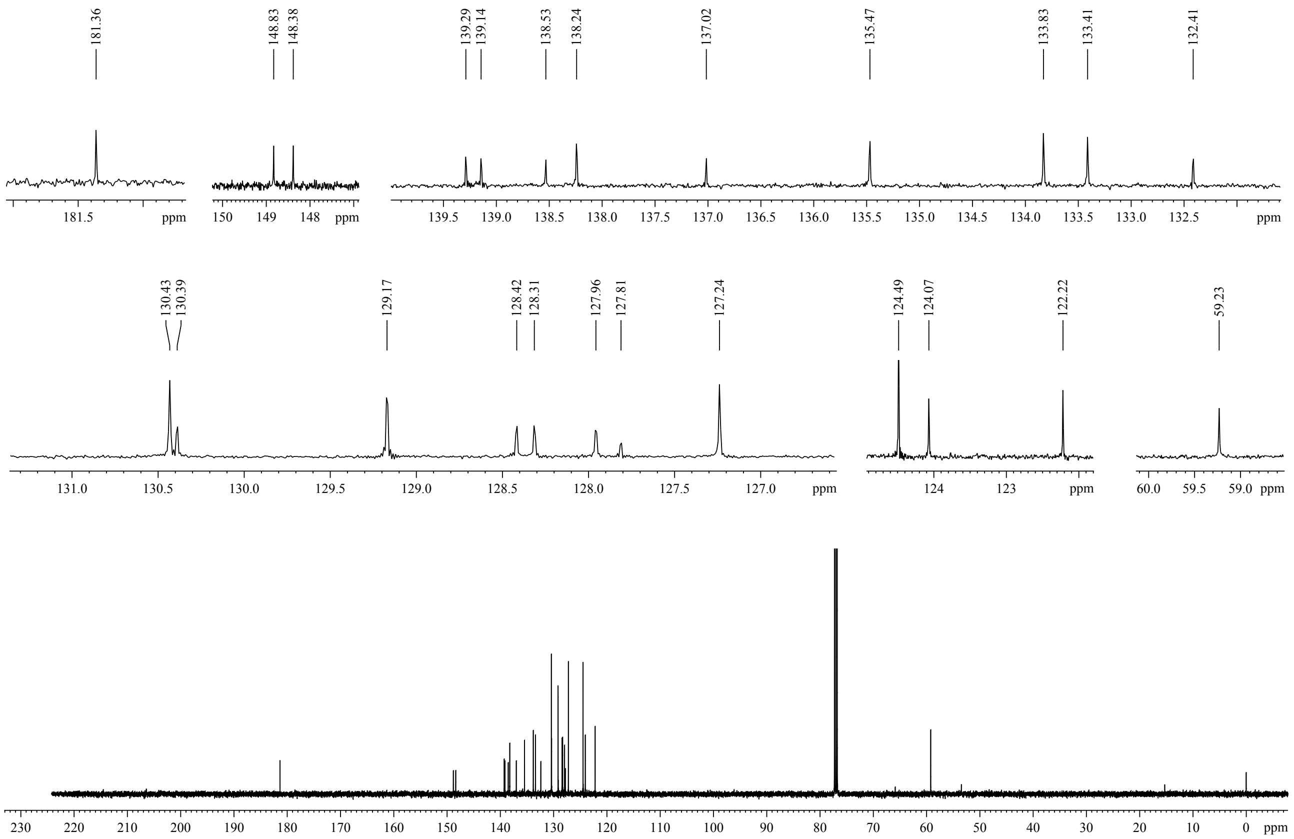

Figure S2. $^{13}C\{^{1}H\}$ NMR spectrum (126 MHz, $CDCl_3$) of compound **QC-NO$_2$**.

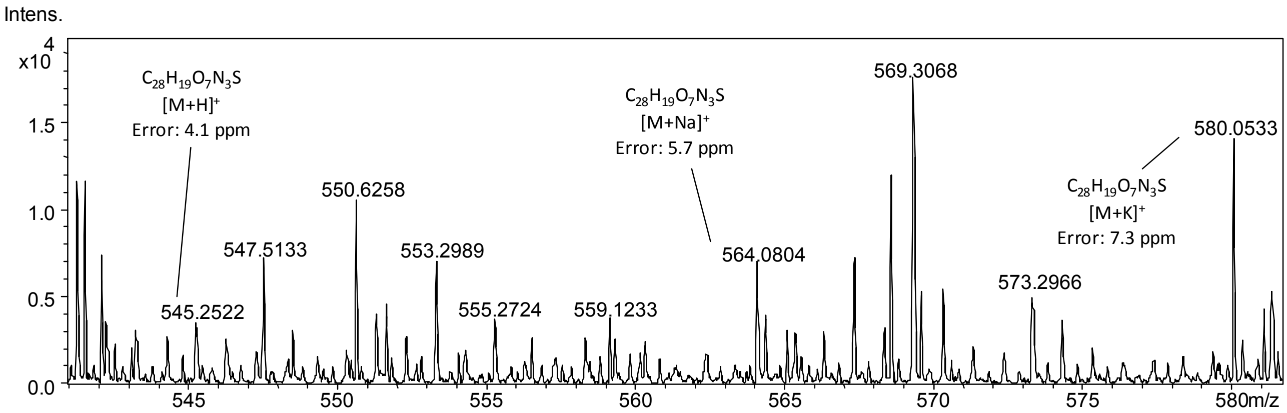

Figure S3. High resolution mass spectrum (HRMS) of compound **$QC\text{-}NO_2$**.

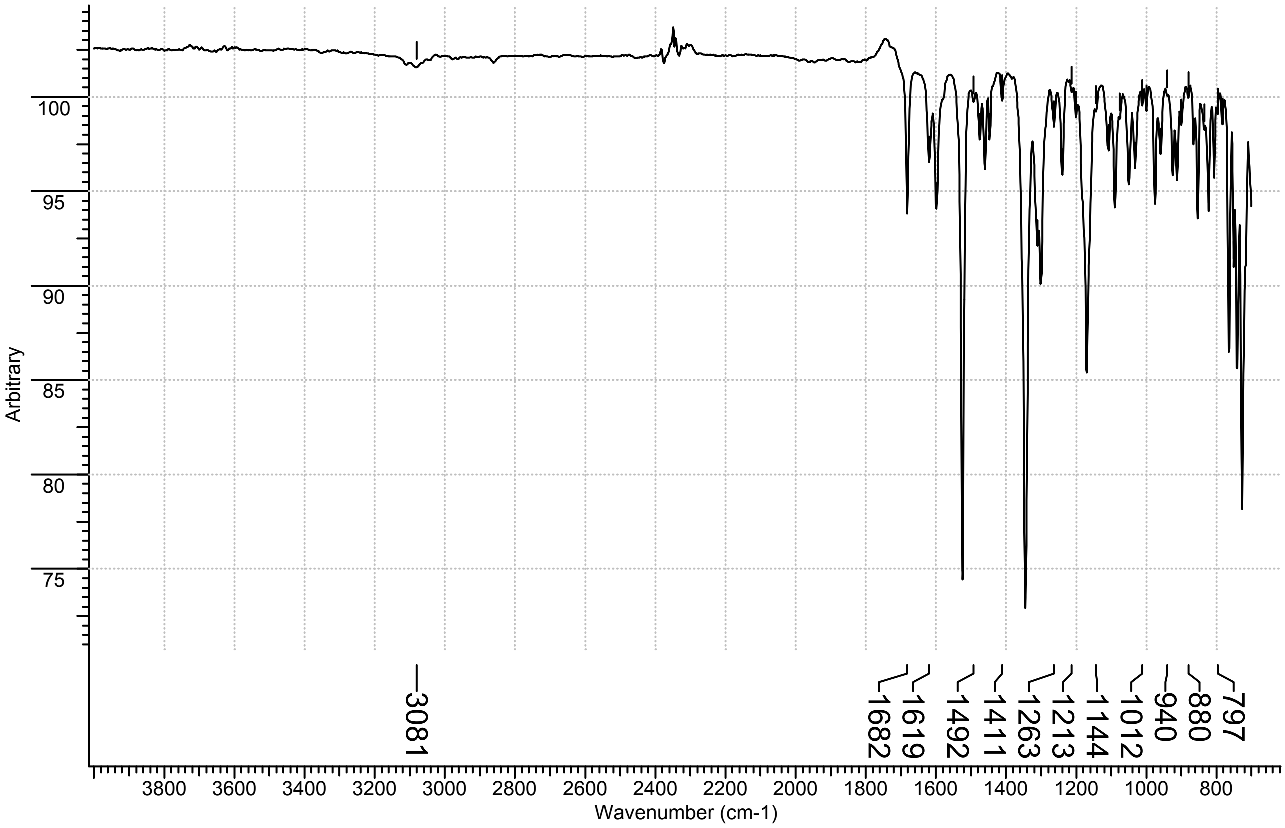

Figure S4. Infrared spectrum (FTIR) of compound **QC-NO_2**.

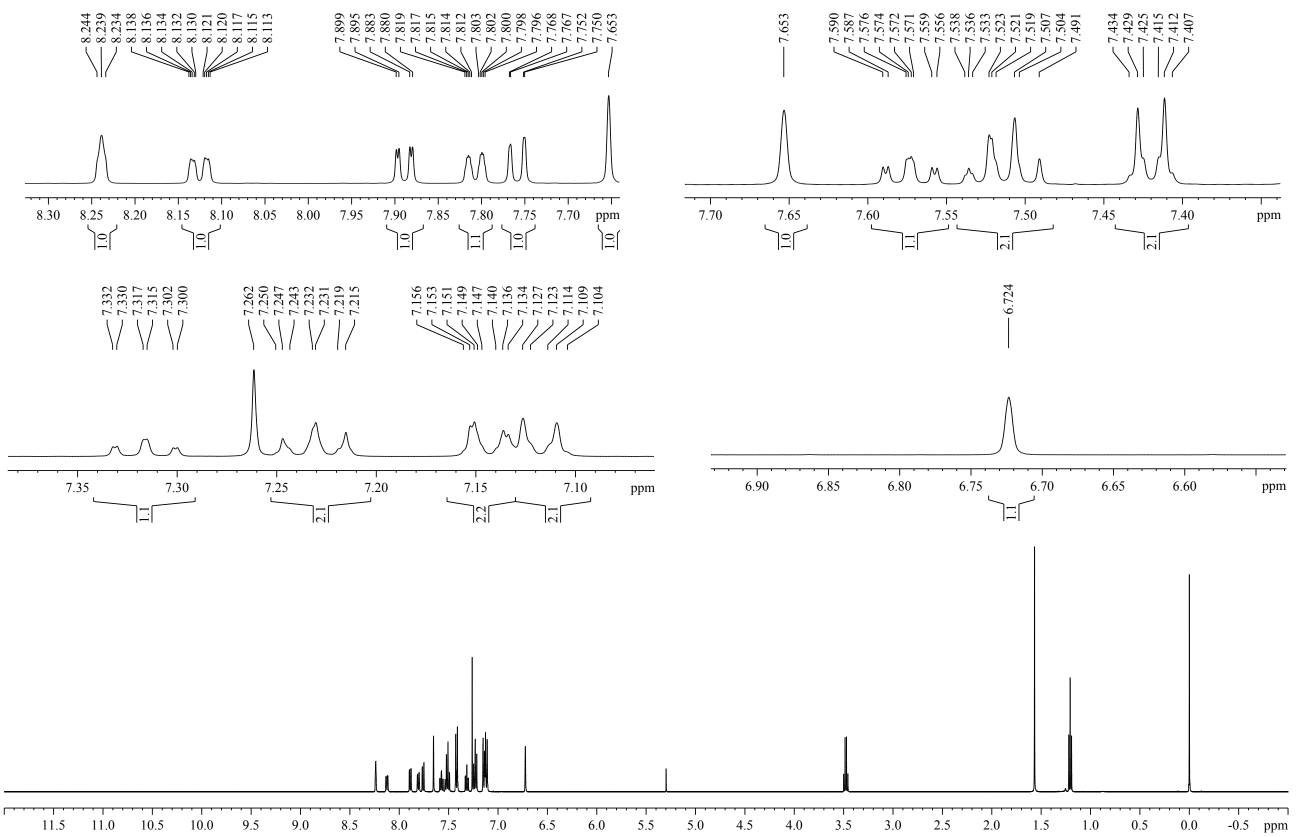

Figure S5. ^{1}H NMR spectrum (500 MHz, $CDCl_3$) of compound **QC-Cl**.

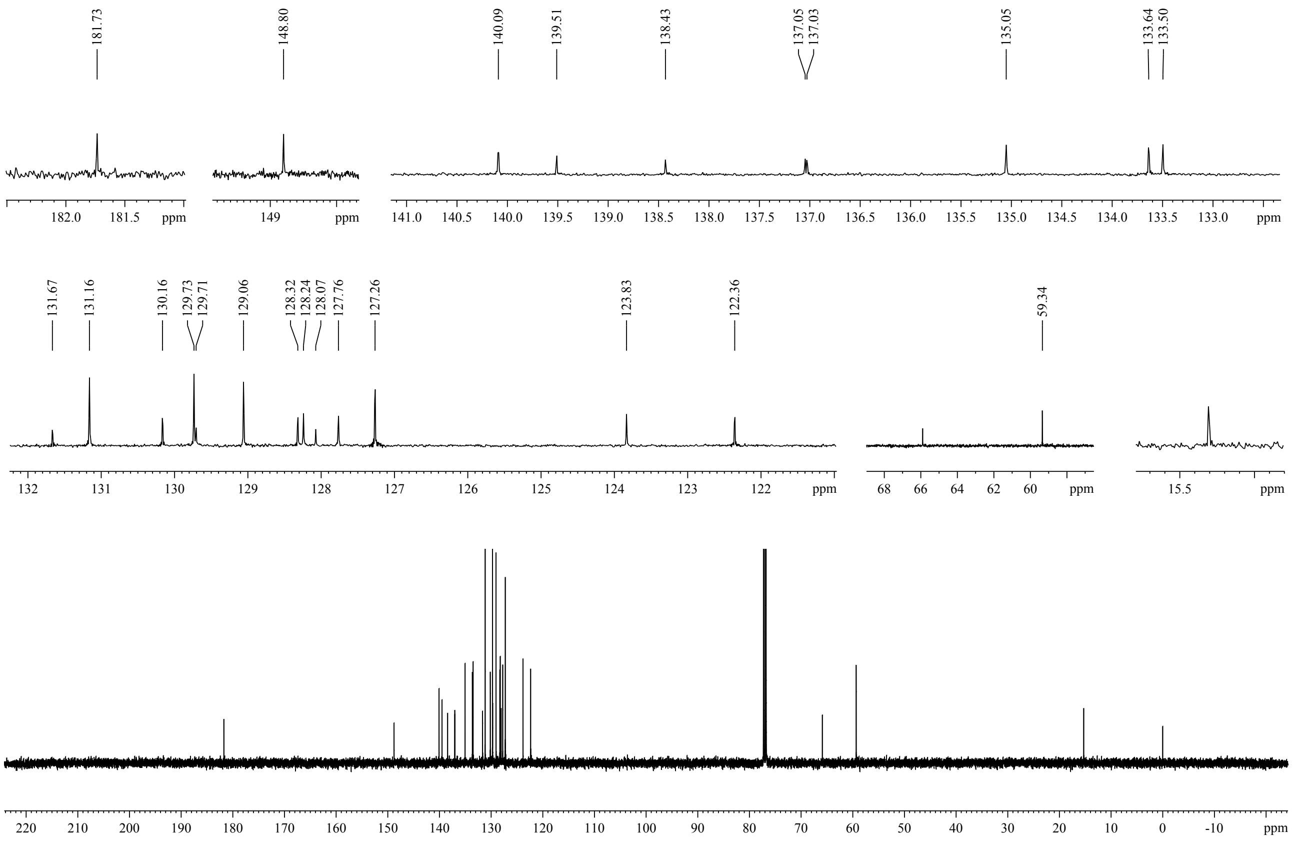

Figure S6. $^{13}C\{^{1}H\}$ NMR spectrum (126 MHz, $CDCl_3$) of compound **QC-Cl**.

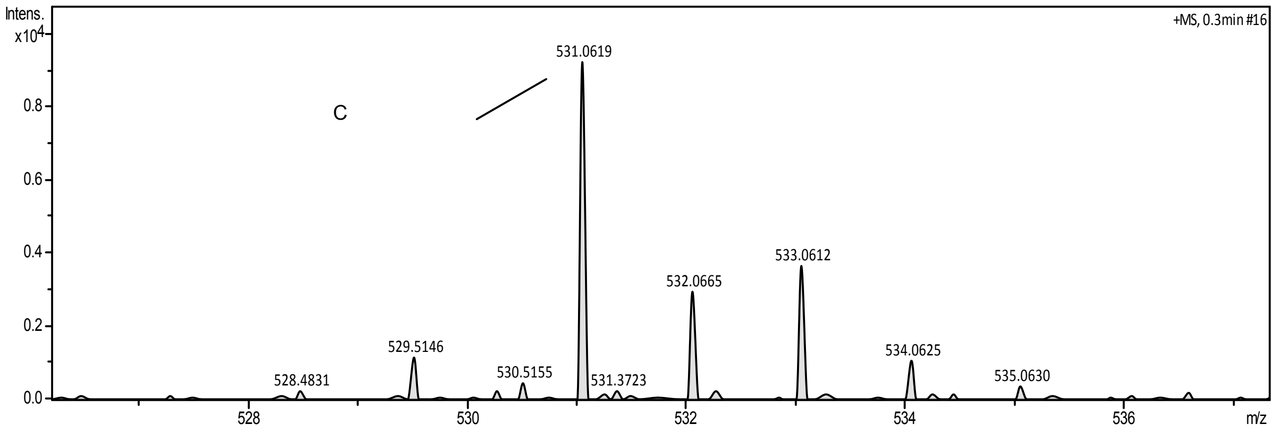

Figure S7. High resolution mass spectrum (HRMS) of compound **QC-Cl**.

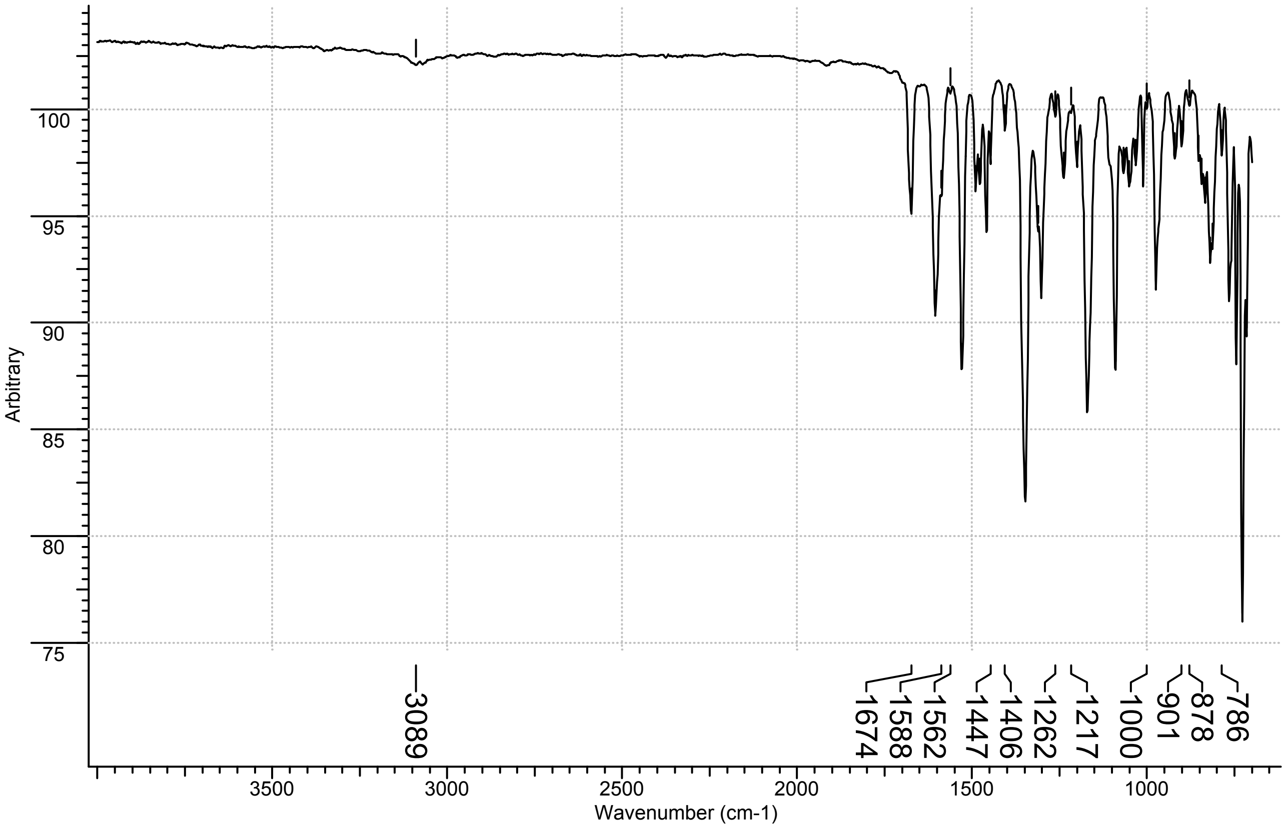

Figure S8. Infrared spectrum (FTIR) of compound **QC-Cl**.

Table S1. Crystallographic data and structure refinement for **QC-NO_2** and **QC-Cl**.

	**QC-NO_2**	**QC-Cl**
Formula	$C_{28}H_{19}N_3O_7S \cdot 0.75(CCl_2)$,0.7C 0.2O	$C_{28}H_{19}ClN_2O_5S \cdot 0.5C_2HO$
Formula weight	615.45 g/mol	571.99 g/mol
Temperature	120(2) K	296.15 K
Wavelength	0.71073 Å	0.71073 Å
Crystal system, space group, Z	Triclinic, $P\bar{1}$, 2	Triclinic, $P\bar{1}$, 2
Unit cell dimensions	a = 10.420(2) Å<br>b = 11.338(3) Å<br>c = 12.447(3) Å<br>α = 74.911(3)°<br>β = 77.293(3)°<br>γ = 71.473(3)°	a = 10.4169(10) Å<br>b = 11.8250(12) Å<br>c = 12.5738(13) Å<br>α = 67.489(3)°<br>β = 77.470(3)°<br>γ = 71.697(3)°
Volume	1331.0(5) $Å^3$	1350.2(2) $Å^3$
Calculated density	1.536 g/cm^3	1.407 g/cm^3
Absorption coefficient	0.330 mm^{-1}	0.267 mm^{-1}
Goodness-of-fit (S)	1.087	1.062
Final R índices [I > 2σ(I)]	R_1 = 0.0709; wR_2 = 0.1927	R_1 = 0.0432; wR_2 = 0.1119
R indices (all data)	R_1 = 0.0898; wR_2 = 0.2043	R_1 = 0.0526; wR_2 = 0.1216
